# Effects of Adding Incentive Spirometry to Hospital‐Based Cardiovascular Rehabilitation on Pulmonary Complications, Hospital Length of Stay, and Clinical‐Functional Recovery After Cardiac Surgery: A Randomized Controlled Trial

**DOI:** 10.1002/pri.70325

**Published:** 2026-08-23

**Authors:** Bruna Elise da Silva Messias, Thamires Alessandra Silveira da Silva, Rafaela Anversa Schreiner, Letícia Torres, Jéssica Bischoff, Taís Flores de Oliveira, Vinicius Peringer, Francine Manara Bortagarai, Mateus Diniz Marques, Bruna Eibel, Carine Cristina Callegaro

**Affiliations:** ^1^ Graduate Program in Human Communication Disorders Federal University of Santa Maria‐UFSM Santa Maria Rio Grande do Sul Brazil; ^2^ Physiology and Rehabilitation Laboratory, Federal University of Santa Maria‐UFSM Santa Maria Rio Grande do Sul Brazil; ^3^ Institute of Cardiology of Porto Alegre‐University Foundation of Cardiology (IC/FUC) Porto Alegre Rio Grande do Sul Brazil; ^4^ Physiotherapy and Rehabilitation Department Federal University of Santa Maria‐UFSM Santa Maria Rio Grande do Sul Brazil

**Keywords:** breathing exercises, cardiac rehabilitation, cardiovascular diseases, incentive spirometry, risk factors, thoracic surgery

## Abstract

**Background and Purpose:**

This study investigated the effects of combining incentive spirometry with cardiac rehabilitation compared with cardiac rehabilitation alone on postoperative pulmonary complications, clinical‐functional recovery, and hospital length of stay in patients undergoing cardiac surgery.

**Methods:**

Randomized controlled trial was conducted from May 2019 to October 2023 in two hospitals, including 46 inpatients undergoing cardiac surgery. Participants were assigned to incentive spirometry plus cardiac rehabilitation or cardiac rehabilitation alone. Both interventions were performed twice daily; spirometry used a volume‐oriented device, and rehabilitation followed a seven‐step protocol (2–4 METs). Outcomes included postoperative pulmonary complications, functional capacity (6‐min walk test), handgrip strength, respiratory muscle function, and length of hospital stay.

**Results:**

The incentive spirometry associated with cardiac rehabilitation group had a longer extracorporeal circulation time (98 ± 26 min) than the cardiac rehabilitation group (76 ± 1; *p* = 0.008). Both groups showed a postoperative decline in respiratory muscle strength, and walking distance (MD: −64.37 m; 95% CI: [−24.1; −104.6]; *d* = 0.71), with no difference in postoperative pulmonary complications and handgrip strength. The incentive spirometry associated with cardiac rehabilitation group did not significantly differ on postoperative hospital stay compared with the cardiac rehabilitation group (MD: −1 day; 95% CI: [−4.71; 2.71]; *d* = −0.19).

**Conclusions:**

In this study, no additional benefit was observed with the addition of incentive spirometry to cardiac rehabilitation compared with cardiac rehabilitation alone. No significant differences were detected between groups in postoperative pulmonary complications, hospital length of stay, or clinical‐functional recovery among individuals undergoing cardiac surgery.

**Trial Registration:**

Brazilian Registry of Clinical Trials (REBEC) under the number RBR‐8tsjf97

## 1 Introduction

Cardiac surgery improves survival, enhances physical exercise capacity, and increases overall quality of life (Borges et al. 2022; Windecker et al. 2014). Advances in surgical techniques and perioperative care have contributed to reduced mortality rates in recent decades (Vervoort 2019). However, the COVID‐19 pandemic significantly reduced the number of elective cardiac surgeries, resulting in longer waiting lists and clinical deterioration among patients. Studies have reported increased postoperative complications and mortality rates of up to 46.7% in unvaccinated individuals undergoing surgery who tested positive for COVID‐19 (Pau et al. 2022; Salenger et al. 2020; Wang et al. 2021).

Postoperative pulmonary complications (PPCs) remain a major cause of morbidity and mortality following cardiac surgery and are associated with prolonged hospital stays and reduced quality of life (Chang et al. 2023; de Moura et al. 2024; Katsura et al. 2015; Kendall et al. 2018). The incidence of PPCs in cardiac surgery ranges from 10% to 25%, with common conditions including atelectasis, pneumonia, pleural effusion, pulmonary edema, respiratory failure, and acute respiratory distress syndrome (de Moura et al. 2024; Katsura et al. 2015; Kendall et al. 2018). These complications contribute substantially to increased healthcare costs and adverse clinical outcomes (Chang et al. 2023; de Moura et al. 2024; Katsura et al. 2015).

Cardiac rehabilitation (CR), which includes respiratory exercises, functional training, and patient education, is an essential component of postoperative management aimed at reducing complications and promoting recovery (Gremeaux and Casillas 2017). Incentive spirometry (IS) is a low‐cost and widely used intervention to prevent PPCs, particularly atelectasis, after major surgery (Chang et al. 2023; Freitas et al. 2012). Although some studies have reported reductions in atelectasis (Sweity et al. 2021), hospital length of stay (Sweity et al. 2021; Eltorai et al. 2019), and duration of mechanical ventilation with IS use (Sweity et al. 2021), other investigations and meta‐analyses have found limited or no additional benefit compared with standard respiratory care (Alaparthi et al. 2021; Manapunsopee et al. 2020; Silva et al. 2024; Sullivan et al. 2021). The overall quality of evidence remains low, highlighting the need for well‐designed clinical studies to clarify the role of IS in perioperative rehabilitation (Sullivan et al. 2021).

Despite the widespread use of IS in clinical practice, evidence regarding its effectiveness when combined with postoperative CR in patients undergoing cardiac surgery remains limited. Therefore, this study aimed to evaluate the effects of IS combined with CR (IS + CR) compared with CR alone on PPCs, hospital length of stay, and clinical‐functional recovery in individuals undergoing cardiac surgery.

## 2 Methods

This multicenter, randomized controlled clinical trial with blinded outcome assessment was conducted between May 2019 and October 2023 in the Coronary Intensive Care Unit and Coronary Ward of the University Hospital of Santa Maria (HUSM), the coordinating center of the study, and in the Postoperative Unit (POU) and the Postoperative Inpatient Unit (B2) of the Institute of Cardiology, University Foundation of Cardiology (IC/FUC), the collaborating center. However, data collection was suspended from March 2020 to March 2023 because of the COVID‐19 pandemic. During this period, all non‐essential research activities were halted, and breathing‐exercise interventions were temporarily prohibited. Recruitment resumed only after the hospital authorized the continuation of research activities and permitted the performance of respiratory exercises. Importantly, none of the participants included in the study were diagnosed with COVID‐19 before or after surgery. This was a three‐arm study consisting of the following groups: IS + CR, CR, and a control group. This manuscript specifically compares the effects of IS + CR versus CR. The sample consisted of 46 patients who were randomized through the website http://www.randomization.com to IS + CR (*n* = 26) or CR (*n* = 20). The randomization list was generated by an independent researcher who was not involved in participant recruitment, evaluations, or intervention delivery. Allocation was concealed until the commencement of the interventions. Outcome assessors remained blinded to treatment allocation throughout the study. Owing to the nature of the intervention, complete participant blinding was not feasible because participants in the incentive spirometry group actively used the device. To minimize expectancy and placebo effects, all participants were informed that the study compared two postoperative rehabilitation protocols without disclosing the specific study hypotheses or any anticipated superiority of one intervention over the other. Likewise, participants were not informed of the specific objectives associated with each intervention. This partial disclosure approach, commonly used in rehabilitation and behavioral intervention research, was adopted to reduce the potential influence of participants' expectations on study outcomes. All patients received physiotherapy solely within the scope of this study, unless complications or clinical instability required additional intervention. Patients aged 40 years or older, admitted to the cardiovascular unit for valve replacement surgery, CABG, or both, were eligible for this study. Exclusion criteria included patients with a prior diagnosis of chronic heart failure, peripheral neuropathy, musculoskeletal disorders, morbid obesity (BMI > 40 kg/m^2^), unstable angina, severe coronary trunk injury, severe valvular disease, nephropathy, infectious diseases, cancer patients (post‐chemotherapy), and those whose clinical condition worsened during hospitalization. All participants signed an informed consent form approved by the Research Ethics Committees prior to enrolling in the study. The study followed the recommendations of the Consort Statement and was approved by the Research Ethics Committees of the Federal University of Santa Maria and the Research Ethics Committees of the Institute of Cardiology/University Foundation of Cardiology (IC/FUC) with report numbers 04444018.2.1001.5346 and 04444018.2.2001.5333, respectively. This study was retrospectively registered in the Brazilian Registry of Clinical Trials (ReBEC) under registration number RBR‐8tsjf97.

Patients underwent evaluations and a preoperative physical therapy session to familiarize themselves with the techniques to be used postoperatively, including diaphragmatic breathing, non‐invasive positive pressure ventilation (NIPPV), positive expiratory pressure mask use, deep breathing exercises, and inspiratory muscle training (IMT). Additionally, they received information about the surgery and postoperative care. Data were collected through anamnesis and tests assessing muscle strength, respiratory function, and functional capacity.

Respiratory muscle strength was measured using a manovacuometer (+300 cmH_2_O to −300 cmH_2_O) (Pereira 2002). Handgrip strength (HGS) was measured using a digital hand dynamometer (Saehan, SH1001, Korean) (Massy‐Westropp et al. 2004). The six‐minute walk test (6 MWT) was conducted according to the recommendations of the American Thoracic Society (ATS Committee 2002).

### 2.1 Preoperative Interventions

Patients in both the IS + CR and CR groups received information about the surgery, including details on the surgical wound, mediastinal and/or thoracic drains, and other access routes, as well as potential impairments in pulmonary function and postoperative pain. All patients were instructed on how to perform an effective cough postoperatively and were familiarized with the visual analog pain scale, to perform IMT (Turky and Afify 2017) and the use of expiratory positive airway pressure and continuous positive airway pressure masks (Barbas et al. 2014). Additionally, those in the IS + CR group received an IS session (Britto et al. 2014). Details about the preoperative interventions are provided in Supporting Information S1.

### 2.2 Postoperative Interventions

CR was divided into seven steps adapted from Winkelmann et al. (2015). All patients received two daily sessions with a trained physiotherapist who was blinded to the evaluations. This continued until hospital discharge or for up to seven postoperative days. If hospitalization extended beyond this period, patients continued receiving conventional care from the hospital unit. CR was progressively applied according to the patient's clinical condition. Cardiac rehabilitation (CR) was divided into seven steps, adapted from the protocol of Winkelmann et al. (2015), and was performed twice daily.

- *Step 1 (2 METs)*—Patient lying in bed. If atelectasis is present, apply noninvasive ventilation (NIV) or positive expiratory pressure (PEP) and perform pulmonary expansion exercises. If pulmonary secretions are present, perform bronchial hygiene maneuvers (flutter, forced expiration technique). Include inspiratory muscle training, active extremity exercises, active‐assisted exercises for the elbows and knees (3 sets of 10 repetitions per exercise), and drain clearance.
- *Step 2 (2 METs)*—Patient seated in bed. If atelectasis is present, apply NIV or PEP and perform pulmonary expansion exercises. If pulmonary secretions are present, perform bronchial hygiene maneuvers (flutter, forced expiration technique). Include inspiratory muscle training, active extremity exercises, and active lower limb exercises (quadriceps) (3 sets of 10 repetitions per exercise), along with drain clearance.
- *Step 3 (3–4 METs)*—Patient standing. If pulmonary secretions are present, perform bronchial hygiene maneuvers (flutter, forced expiration technique). Include inspiratory muscle training, active exercises for the elbows, knees, and extremities, and active stretching of the lower limbs (quadriceps, adductors, hamstrings, and triceps) (3 sets of 10 repetitions per exercise). Perform stationary marching for 2 minutes and walk 35 m.
- *Step 4 (3‐4 METs)*—Patient standing. If pulmonary secretions are present, perform bronchial hygiene maneuvers (flutter, forced expiration technique). Include inspiratory muscle training, active extremity exercises, and active lower limb exercises (flexion, extension, abduction, and adduction) (3 sets of 10 repetitions per exercise). Walk 40–60 m and descend one flight of stairs.
- *Step 5 (3–4 METs)*—Patient standing. If pulmonary secretions are present, perform bronchial hygiene maneuvers (flutter, forced expiration technique). Include inspiratory muscle training, active extremity exercises, and active lower limb exercises (flexion, extension, abduction, and adduction) (3 sets of 10 repetitions per exercise). Walk 100–150 m and descend and ascend one flight of stairs.
- *Step 6 (3–4 METs)*—Patient standing. If pulmonary secretions are present, perform bronchial hygiene maneuvers (flutter, forced expiration technique). Include inspiratory muscle training, active extremity exercises, and active lower limb exercises (flexion, extension, abduction, and adduction) (3 sets of 10 repetitions per exercise). Walk 150–200 m and descend and ascend two flights of stairs.
- *Step 7 (3–4 METs)*—Patient standing. If pulmonary secretions are present, perform bronchial hygiene maneuvers (flutter, forced expiration technique). Include inspiratory muscle training, active extremity exercises, and active lower limb exercises (flexion, extension, abduction, and adduction) (3 sets of 10 repetitions per exercise). Walk 200 m and descend and ascend three flights of stairs.

Progression through the steps depended on patient tolerance. If heart rate increased by more than 20 beats per minute or signs of intolerance appeared, the patient returned to the previous step. Postoperative techniques were taught preoperatively, and inspiratory muscle training began 1–24 hours after extubation, as determined by the physical therapy team. The protocol continued until postoperative day 7 or hospital discharge.

The IS + CR group received IS, as described in the preoperative interventions, combined with CR.

### 2.3 Postoperative Assessments

Inspiratory muscle strength was reassessed on postoperative days 1 (if extubated; otherwise, assessments were performed after extubation), 3, 5, and 7, or at hospital discharge if earlier than day 7. Functional capacity and handgrip strength were measured on postoperative day 7 or at discharge if earlier. Data regarding the duration of invasive mechanical ventilation, length of hospital stay, and postoperative complications—including fever, atrial fibrillation, and PPCs such as pneumonia, atelectasis, pleural effusion, and pulmonary edema—were collected from medical records. PPC diagnoses were established by the attending medical team according to routine institutional clinical practice.

### 2.4 Data Analysis

To detect a minimum difference of 50 m in the 6MWT, with an alpha error of 5% and a power of 80%, the minimum required sample size was calculated to be 12 individuals per group, totaling 24 patients, based on a previous study (Jacob et al. 2023). Descriptive data were presented as means and standard deviations or as absolute and relative frequencies. Normality was assessed using the Shapiro‐Wilk test. Comparisons of clinical and sociodemographic characteristics, surgical data, postoperative complications, and hospital length of stay between intervention groups were conducted using the Chi‐square test (categorical variables), Student's *t*‐test for independent samples (continuous variables), or the Mann‐Whitney U test (continuous variables), depending on data normality.

Analyses were conducted according to the per‐protocol principle, including only participants who completed the allocated intervention and underwent postoperative outcome assessments. Participants who lost the follow‐up due to postoperative complications or who did not complete the postoperative assessments were excluded from the final analyses. To analyze the effect of the intervention over time (preoperative, postoperative days 1 and 3, and hospital discharge), Generalized Estimating Equations (GEE) were used (Zeger et al. 1988). The same test was applied to assess intervention effects, time effects (preoperative, postoperative days 1 and 3, and hospital discharge), and interaction effects (intervention × time) on manovacuometry parameters (MIP, %MIP, MEP, and % MEP). Bonferroni post hoc tests were applied when necessary. All analyses were performed using SPSS Statistics 26, with a significance level of *p* < 0.05.

## 3 Results

**FIGURE 1:**
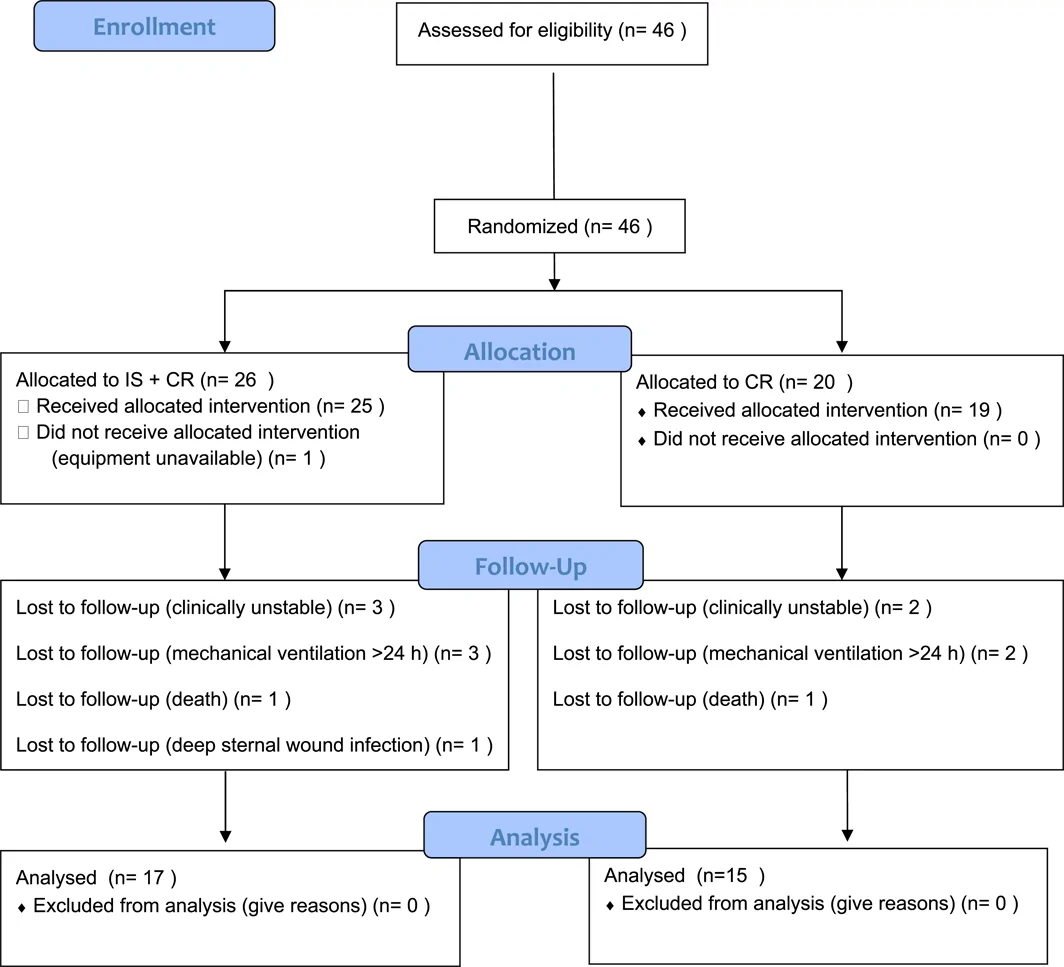
Study flowchart.

A total of 46 individuals were randomized, with 26 assigned to the IS + CR group and 20 to the CR group. Both groups experienced follow‐up losses, related to surgical complications, resulting in a final sample of 32 individuals, as shown in Figure 1. No adverse events or harms related to IS + CR or CR group were observed during the intervention period. All sessions were conducted under physiotherapist supervision and were interrupted if any signs of discomfort or clinical instability occurred; however, no such events were recorded.

The clinical and sociodemographic characteristics are presented in Table 1. The IS + CR and CR groups did not differ in terms of sex, age, or cardiovascular risk factors. Additionally, no significant differences were observed between the groups in preoperative respiratory muscle strength.

**TABLE 1:** Clinical and sociodemographic characteristics.

	CR (*n* = 15)	IS + CR (*n* = 17)	*p*
Sex			
Female, % (*n*)	27 (4)	18 (3)	0.678
Male, % (*n*)	73 (11)	82 (14)	
Age (years)	60.27 ± 7.6	64.27 ± 8.5	
Weight (kg)	77.83 ± 12.4	76.83 ± 19.2	
BMI (kg/m^2^)	27.44 ± 3.8	26.71 ± 4.9	
Type of heart disease			
Ischemic, % (*n*)	87 (13)	59 (10)	0.122
Valvular, % (*n*)	13 (2)	41 (8)	
SAH, % (*n*)	80 (12)	76 (13)	1.0
DM 1, % (*n*)	7 (1)	6 (1)	1.0
DM 2, % (*n*)	40 (6)	29 (5)	0.72
Physical inactivity, % (*n*)	60 (9)	53 (9)	0.735
Arrhythmias, % (*n*)	7 (1)	29 (5)	0.178
Obesity, % (*n*)	27 (4)	12 (2)	0.383
Previous cardiac surgery, % (*n*)	20 (3)	6 (1)	0.319
Dyslipidemia, % (*n*)	27 (4)	29 (5)	1.0
Smoking, % (*n*)	27 (4)	18 (3)	0.678
Former smoker, % (*n*)	47 (7)	53 (9)	1.0
COPD, % (*n*)	7 (1)	6 (1)	1.0
MI < 90 days, % (*n*)	47 (7)	35 (6)	0.720
Rest angina, % (*n*)	27 (4)	12 (2)	0.383
Active endocarditis, % (*n*)	0	6 (1)	1.0
Functional classification (NYHA)			
NYHA I, % (*n*)	27 (4)	29 (5)	0.678
NYHA II % (*n*)	7 (1)	24 (4)	
NYHA III, % (*n*)	7 (1)	6 (1)	
NYHA IV, % (*n*)	7 (1)	0	
Respiratory muscle strength			
MIP (cmH_2_O)	79 ± 32.17	74 ± 39.46	0.688
% predicted MIP	80 ± 33.64	73 ± 35.44	0.618
MEP (cmH_2_O)	99 ± 43.43	95 ± 54.32	0.808
% predicted MEP	93 ± 38.14	87 ± 44.87	0.690

*Note:* The data are presented as mean (± SD) and % (*n*).

Abbreviations: BMI, body mass index; COPD, chronic obstructive pulmonary disease; DM, diabetes mellitus type 1 or 2; IS + CR, incentive spirometry combined with cardiac rehabilitation; MEP, maximum expiratory pressure; MI, myocardial infarction; MIP, maximum inspiratory pressure; NYHA, New York Heart Association; PO, postoperative; RC, cardiac rehabilitation; SAH, systemic arterial hypertension.

*p < 0.05.

Table 2 presents data on surgery, postoperative complications, and hospital length of stay. The IS + CR group exhibited a longer extracorporeal circulation time compared to the CR group, with a large effect size (MD: 22 min; 95% CI: [7.96; 36.04]; *d* = 1.11). No significant differences were observed between groups in preoperative hospital stay (MD: −2 days; 95% CI: [−8.02; 4.02]; *d* = −0.23), postoperative hospital stay (MD: −1 day; 95% CI: [−4.71; 2.71]; *d* = −0.19), or total hospitalization time (MD: −2 days; 95% CI:[−10.37; 6.37]; *d* = −0.18). Similarly, the incidence of postoperative complications did not differ significantly between groups.

**TABLE 2:** Surgical data, postoperative complications, and hospital length of stay.

	CR (*n* = 15)	IS + CR (*n* = 17)	*p*
Type of surgery			
CABG, % (*n*)	87 (13)	53 (9)	0.73
VRS, % (*n*)	13 (2)	35 (6)	
CABG + VRS, % (*n*)	0	12 (2)	
ECT, % (*n*)	93 (14)	100 (17)	0.469
Surgery time (min)	207 + 37	215 ± 24	0.593
ECT time (min)	76 ± 11	98 ± 26	0.008*
Pneumonia, % (*n*)	7 (1)	18 (3)	0.603
Atelectasis, % (*n*)	40 (6)	18 (3)	0.243
Pleural effusion, % (*n*)	40 (6)	53 (9)	0.502
Fever, % (*n*)	20 (3)	41 (7)	0.265
Atrial fibrillation, % (*n*)	13 (2)	18 (3)	1.0
Pulmonary edema, % (*n*)	20 (3)	18 (3)	1.0
Preoperative hospitalization time (days)	15 ± 7.9	13 ± 9.21	0.688
Postoperative hospitalization time (days)	10 ± 6.78	9 ± 2.73	0.678
Total hospitalization time (days)	24 ± 13.36	22 ± 9.56	0.629

*Note:* Data are presented as mean (± SD) and % (*n*).

Abbreviations: CABG, coronary artery bypass grafting; CR, Cardiac Rehabilitation; ECT, extracorporeal circulation time; IS + CR, incentive spirometry combined with cardiac rehabilitation; VRS, valve replacement surgery.

^*^ *p* < 0.05.

The duration of extracorporeal circulation did not significantly differ according to the occurrence of postoperative complications. Mean extracorporeal circulation time did not differ between patients with and without postoperative pneumonia (98.0 ± 13.9 min vs. 92.9 ± 29.5 min, *p* = 0.73), atelectasis (95.6 ± 34.2 min vs. 92.7 ± 25.8 min, *p* = 0.79), pulmonary edema (88.8 ± 23.7 min vs. 94.6 ± 29.1 min, *p* = 0.65), fever (94.6 ± 24.5 min vs. 93.0 ± 29.9 min, *p* = 0.88), atrial fibrillation (91.2 ± 29.4 min vs. 94.0 ± 28.2 min, *p* = 0.84), or pleural effusion (96.8 ± 32.4 min vs. 90.4 ± 23.5 min, *p* = 0.53). In addition, exploratory analyses showed no significant associations between extracorporeal circulation time and preoperative hospital length of stay (*ρ* = −0.05, *p* = 0.77), postoperative hospital length of stay (*ρ* = 0.20, *p* = 0.25), or total hospital length of stay (*ρ* = −0.05, *p* = 0.77).

**FIGURE 2:**
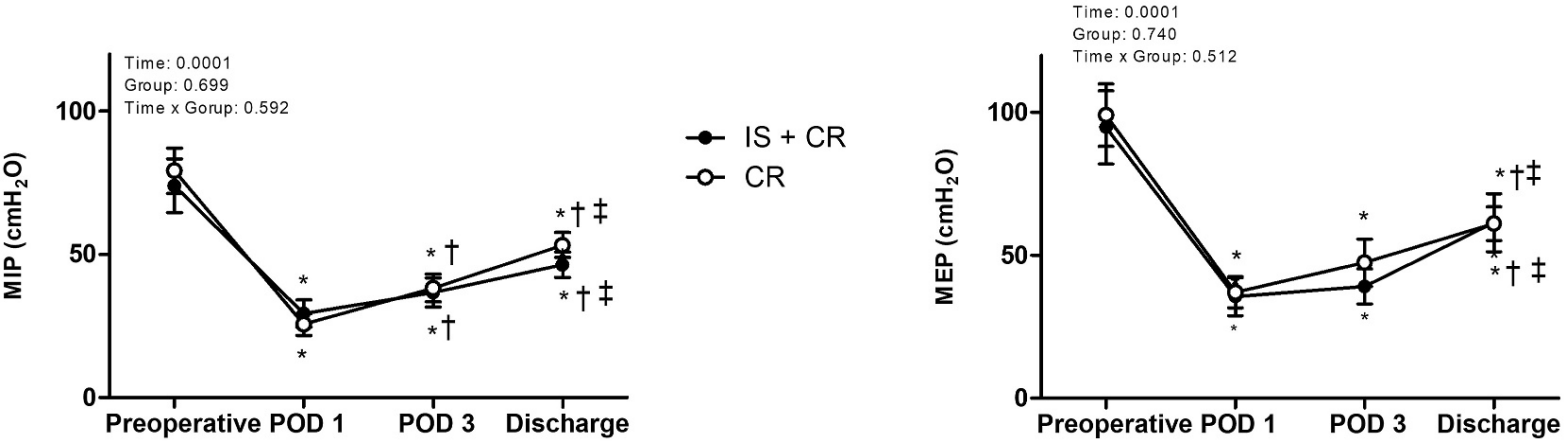
Respiratory muscle strength data are presented as mean ± standard deviation (SD). Results of the repeated‐measures analysis are presented within each panel (time effect, group effect, and time × group interaction). CR = cardiac rehabilitation; IS + CR = incentive spirometry combined with cardiac rehabilitation; MEP = maximum expiratory pressure; MIP = maximum inspiratory pressure; POD = postoperative day. Statistical symbols indicate within‐group comparisons: *p < 0.05 versus preoperative assessment; †*p* < 0.05 versus POD 1; ‡*p* < 0.05 versus POD 3.

Respiratory muscle strength is presented in Figure 2. Inspiratory and expiratory muscle strength decreased from the first postoperative day (POD) until hospital discharge compared with preoperative values in both groups. Inspiratory muscle strength progressively increased from the third POD to hospital discharge compared with the first POD but did not return to preoperative values in either group. Expiratory muscle strength increased only at hospital discharge, without returning to preoperative values in both groups.

Table 3 presents the clinical progression of the groups. During the seven postoperative days, no significant differences were observed in group progression. The effects of CR and IS + CR on the 6MWT and handgrip strength are presented in Table 4. A significant, moderate‐to‐large reduction in functional capacity was observed following surgery (MD: −64.37 m; 95% CI: [−24.1; −104.6]; *d* = 0.71). Furthermore, the difference in change between groups (interaction effect) was negligible and not statistically significant (MD: 6.23 m; 95% CI: [−74.2; 86.6]; *d* = 0.07). A generalized estimating equations (GEE) model with an independent working correlation structure and robust standard errors was used to evaluate the effects of time and intervention group on the 6MWD, adjusting for extracorporeal circulation time. The analysis revealed a significant main effect of time, no significant main effect of intervention group, and the time‐by‐group interaction, indicating that the magnitude of change in 6MWD over time was similar between the intervention groups. The covariate extracorporeal circulation time was not significantly associated with 6MWD (Wald *χ* ^2^ = 0.022, *p* = 0.882; *β* = −0.114, 95% CI: −1.618 to 1.389), suggesting that baseline extracorporeal circulation time did not influence the functional capacity outcome. Overall, adjustment for extracorporeal circulation time did not materially alter the estimated effects of time or intervention group or the time‐by‐group interaction.

**TABLE 3:** Clinical progression of the groups.

		POD 1	POD 2	POD 3	POD 4	POD 5	POD 6	POD 7
STEP 1	IS + CR % (*n*)	76 (13)	23 (4)	23 (4)	0.5 (1)	12 (2)	0.5 (1)	
	CR % (*n*)	67 (10)	13 (2)		0.5 (1)		0.5 (1)	
STEP 2	IS + CR % (*n*)	17 (3)	53 (9)	12 (2)	17 (3)	0.5 (1)	12 (2	0.5 (1)
	CR % (*n*)	0.5 (1)	67 (10)	33 (5)	20 (3)	0.5 (1)		
STEP 3	IS + CR % (*n*)		0.5 (1)	53 (9)	53 (9)	23 (4)	23 (4)	23 (4)
	CR % (*n*)		13 (2)	47 (7)	26 (4)	26 (4)	0.5 (1)	
STEP 4	IS + CR % (*n*)		12 (2)		0.5 (1)	23 (4)	0.5 (1)	
	CR % (*n*)			20 (3)	13 (2)	0.5 (1)	20 (3)	20 (3)
STEP 5	IS + CR % (*n*)			12 (2)	12 (2)	17 (3)	23 (4)	12 (2)
	CR % (*n*)				0.5 (1)	26 (4)	0.5 (1)	13 (2)
STEP 6	IS + CR % (*n*)				0.5 (1)	17 (3)	12 (2)	12 (2)
	CR % (*n*)				13 (2)	0.5 (1)	0.5 (1)	
STEP 7	IS + CR % (*n*)						0.5 (1)	
	CR % (*n*)					13 (2)		0.5 (1)
*p*		0.624	0.823	0.542	0.678	0.281	0.850	0.258

*Note:* Data are presented as mean (± SD).

Abbreviations: CR, cardiac rehabilitation; IS + CR, incentive spirometry combined with cardiac rehabilitation; POD, postoperative day; Pre‐op, preoperative.

*p* < 0.05.

**TABLE 4:** Effects of CR and IS + CR on functional clinical recovery.

	CR (*n* = 15)	IS + CR (*n* = 17)	*p* within‐group	*p* between‐group	*p* interaction
6MWT					
Distance PreOp (*m*)*	374 ± 29	352 ± 22	0.002*	0.63	0.87
Distance on discharge (*m*)*	307 ± 22	291 ± 43			
Handgrip strength					
Right PreOp (kgf)	39 ± 4.73	35 ± 4.73	0.55	0.403	0.77
Right PO (kgf)	35 ± 4.62	29 ± 3.70			
Left PreOp (kgf)	37 ± 5.69	31 ± 4.92	0.135	0.354	0.943
Left PO (kgf)	34 ± 5.94	27 ± 3.37			

*Note:* Data are presented as mean (± SD) or *(± SE).

Abbreviations: 6MWT, 6‐min walk test; CR, cardiac rehabilitation; IS + CR, incentive spirometry combined with cardiac rehabilitation; PO, postoperative; PreOp, preoperative.

^*^ *p* < 0.05.

No difference in handgrip strength was found after surgery in either group.

## 4 Discussion

This study evaluated the effects of IS + CR compared with CR alone in patients undergoing cardiac surgery. No significant differences were observed between groups in postoperative pulmonary complications, functional recovery, or hospital length of stay; however, the IS + CR group had a longer extracorporeal circulation time. These findings suggest that IS may not provide additional benefits when delivered alongside a structured rehabilitation program.

Previous studies have associated prolonged extracorporeal circulation time (ECC) with increased postoperative morbidity, mortality, and organ dysfunction (Chalmers et al. 2014; Velho et al. 2024). In the present study, the IS + CR group had a significantly longer ECC duration (98 ± 26 min) than the CR group (76 ± 11 min), with a greater proportion of patients exceeding 100 minutes, suggesting a higher baseline surgical risk profile and potentially greater surgical complexity. However, sensitivity analyses adjusting for ECC duration did not materially alter the estimated treatment effects, and ECC was not independently associated with the functional outcome. Nevertheless, given the established relationship between prolonged ECC and adverse postoperative outcomes, as well as the limited sample size of the present study, residual confounding cannot be completely excluded. Therefore, these findings should be interpreted with caution and confirmed in larger randomized trials.

The literature is heterogeneous regarding the benefits of IS. Some studies report reduced respiratory complications and shorter hospital stays with IS (Chang et al. 2023; Eltorai et al. 2019), particularly among patients with impaired baseline pulmonary function (Chang et al. 2023), while systematic reviews and meta‐analyses have found no consistent effect of IS on pulmonary complications or mortality (Sullivan et al. 2021). Our results align with the broader reviews that did not demonstrate an additional benefit of IS when combined with a comprehensive rehabilitation program. Consistent with these latter findings, the present study did not demonstrate significant differences between groups in postoperative pulmonary complications.

A volume‐oriented device was used in this trial. Although some studies suggest the superiority of volume‐oriented over flow‐oriented devices in specific settings (Alaparthi et al. 2021), we found no differences between groups in respiratory muscle strength (MIP/MEP) or functional capacity. A likely explanation is that both groups received a comprehensive postoperative protocol—including inspiratory muscle training, breathing exercises, positive‐pressure techniques (PEP and CPAP/NIV when indicated), airway clearance maneuvers, and progressive mobilization—which may have reduced the ability to detect an incremental effect of IS. Therefore, our findings should be interpreted as indicating that the addition of incentive spirometry did not provide measurable benefits beyond those achieved with a comprehensive physiotherapy program, rather than implying that incentive spirometry is ineffective as a standalone intervention or in different clinical settings.

Regarding respiratory muscle strength, both MIP and MEP decreased on the first postoperative day relative to preoperative values in both groups. MIP gradually improved from the third postoperative day until discharge but did not return to preoperative levels; MEP increased only at hospital discharge. This pattern may reflect the impact of inspiratory muscle training (Xiang et al. 2023) applied to both groups in addition to the natural course of postoperative recovery (Chang et al. 2023; Sweity et al. 2021; Manapunsopee et al. 2020).

The findings of this study, particularly the non‐significant between‐group differences, should be interpreted with caution. Although the a priori sample size calculation indicated adequate statistical power for the primary outcome (6‐min walk test), follow‐up losses substantially reduced the final sample size. Consequently, the study may have been underpowered to detect clinically meaningful differences in postoperative pulmonary complications, hospital length of stay, and functional recovery outcomes, increasing the risk of type II error. Furthermore, analyses were performed according to the per‐protocol principle rather than intention‐to‐treat, which may have increased the risk of attrition bias and reduced the benefits of randomization. The prolonged data collection period and the interruption caused by COVID‐19‐related restrictions should also be considered when interpreting the results, as prolonged waiting times and reduced physical activity during the pandemic may have adversely affected patients' baseline functional status. PPC diagnoses were established by the attending medical teams according to routine institutional clinical practice. As the study was conducted in two hospitals and no standardized research‐specific diagnostic criteria were prospectively applied, some variability in the diagnosis and reporting of individual pulmonary complications may have occurred. In this study, eight participants were not medically cleared to undergo the six‐minute walk test before surgery, and two participants were not medically cleared to undergo the test after surgery. Future randomized controlled trials with larger sample sizes are warranted to confirm these findings and provide more precise estimates of treatment effects.

### 4.1 Implications for Physiotherapy Practice

In patients undergoing cardiac surgery, the routine addition of incentive spirometry to a structured cardiac rehabilitation program may not confer additional clinical benefits in terms of postoperative pulmonary complications, functional recovery, or length of hospital stay. These findings support prioritizing comprehensive, early, and progressive rehabilitation protocols as the cornerstone of physiotherapy care. The use of IS may be reconsidered as a selective measure guided by individual patient needs, risk profile, and clinical presentation rather than as a standardized adjunct.

## 5 Conclusions

This study showed that IS + CR compared with CR alone may not reduce PPCs, hospital length of stay, or improve clinical‐functional recovery in individuals undergoing cardiac surgery. These findings suggest that IS may not provide additional benefits when delivered alongside a structured rehabilitation program. Future randomized controlled trials with larger sample sizes are warranted to confirm these findings.

## Funding

PIC/EBSERH (Grant 01/2022), from the Scientific Initiation Program of the Brazilian Company of Hospital Services (PIC/EBSERH), awarded to Rafaela Anversa Schreiner.

## Ethics Statement

The study was approved by the Research Ethics Committees of the Federal University of Santa Maria and the Research Ethics Committee of the Institute of Cardiology of Porto Alegre‐University Foundation of Cardiology (IC/FUC) with report numbers 04444018.2.0000.5346 and 04444018.2.2001.5333, respectively.

## Consent

All participants signed an informed consent form approved by the Research Ethics Committees prior to enrolling in the study.

## Conflicts of Interest

The authors declare no conflicts of interest.

## Supporting information


Supporting Information S1

## Data Availability

Research data are not shared.
