# Supplementary material for: Effects of Adding Incentive Spirometry to Hospital‐Based Cardiovascular Rehabilitation on Pulmonary Complications, Hospital Length of Stay, and Clinical‐Functional Recovery After Cardiac Surgery: A Randomized Controlled Trial
Source: Physiother Res Int. 2026 Aug 23;31(4):e70325. doi: 10.1002/pri.70325 (PMC13500033; doi:10.1002/pri.70325)
Supplement: Supplementary file 1 [file PRI-31-e70325-s001.docx]

**Preoperative Interventions**

**Inspiratory Muscle Training (IMT)**

Preoperative IMT was performed using a threshold-loaded inspiratory resistance device (PowerBreathe Plus, PowerBreathe International, Warwickshire, UK). In a seated position, patients performed IMT at 30% of their maximal inspiratory pressure (MIP). Resistance was incrementally adjusted based on the patient's perceived effort (PPE) using the Borg scale. If PPE was below 5, resistance was increased by 2 cmH₂O. Resistance was maintained when PPE was rated between 6 and 8 or reduced by 1–2 cmH₂O for PPE levels between 9 and 10. Patients were encouraged to complete three sets of ten slow maximal inspirations, with a 30–60 second rest between sets, performed twice daily.^30^

**Expiratory Positive Airway Pressure (EPAP)**

EPAP was indicated for bronchial secretion clearance, atelectasis prevention or treatment, and reduction of air trapping in patients with asthma or COPD. The procedure was performed with the patient in a seated position, maintaining an inspiration-to-expiration ratio of 1:3 or 1:4. Pressure levels were incrementally adjusted based on the patient’s PPE using the Borg scale. If PPE was below 5, resistance was increased by 5 cmH₂O. Resistance was maintained for PPE values between 6 and 8 and reduced by 5 cmH₂O for PPE values between 9 and 10. The recommended duration was 15 minutes or as tolerated by the patient.

**Continuous Positive Airway Pressure (CPAP)**

CPAP was administered according to the Brazilian guidelines for non-invasive mechanical ventilation from the Brazilian Association of Intensive Medicine’s Mechanical Ventilation Committee and the Brazilian Thoracic Society’s Intensive Therapy Commission^31^. It was delivered through an oronasal mask, with a set pressure of 10 cmH₂O for all patients, applied for a maximum of 40 minutes, twice daily.

**Incentive Spirometry (IS)**

Inspiratory spirometry (IS) was performed using a volume-oriented device (Voldyne) designed to encourage sustained maximal inspiration through visual feedback, thereby preventing or reversing alveolar collapse. Standardized instructions were provided by a physiotherapist to ensure correct technique and patient cooperation. Patients were instructed to seal their lips firmly around the mouthpiece to prevent air leakage, keep the spirometer upright within their visual field, and perform slow, deep inspirations mimicking a sigh. Inspiration was initiated from functional residual capacity and continued steadily until reaching the maximal attainable inspiratory volume, as indicated on the device’s visual scale. No fixed numerical volume target was imposed; instead, patients were encouraged to achieve their maximal inspiratory capacity according to individual lung function and clinical status. After reaching the target volume, patients held their breath for 3–5 seconds before exhaling gently and smoothly back to functional residual capacity without forced expiration. ^24^ Each session consisted of three sets of ten deep inspirations, with a 30–60 second rest interval between sets, performed twice daily. Adherence to the intervention was monitored through direct supervision during in-hospital sessions and daily documentation of completed sessions by the physiotherapy team; any difficulties performing the technique or inability to complete a session were recorded in the clinical chart. IS was performed only when patients were hemodynamically stable and able to cooperate. Sessions were interrupted in the presence of pain, dizziness, oxygen desaturation, hemodynamic instability, or significant discomfort. No adverse events related to IS were observed.
